# Dentists as Bookkeepers and Collectors

**Published:** 1900-12-15

**Authors:** George E. Zinn

**Affiliations:** Elkhart, Ind.


### DENTISTS AS BOOKKEEPERS AND COLLECTORS.

BY DR. GEORGE E. ZINN, ELKHART, IND.

Read at the Union Meeting of the Northern Indiana and Southwestern Michigan Dental Association.

It was due appreciation of the form of the invitation which I received from your Program Committee to write a *short* paper for this meeting that induced me to undertake to put before you a paper that would be worth your time and attention. Your committee was also kind enough to suggest a topic, and after due consideration I was convinced that no other subject of so much concern to dentists was so literally and shamefully slighted as the business end of a dental practice. Like the business end of a bee, for some reason we leave it alone. Indeed, in all the dental meetings I ever attended, and in my experience in two dental colleges, I never heard a lecture, a paper or a discussion on the best way to keep the daily records and accounts of a dental practice. Further than that, in searching files of two leading dental journals for upwards of six years, I found not one article directly on that subject. Nevertheless we must and do keep records and accounts, but how do we do it? There is no method universally followed, but each dentist seems to have a system of his own, and the different systems for this work are probably as numerous as for any other particular proceeding of our practice.

The only thing that is an attempt to unify this part of our work is the commercial enterprise of a few private individuals who have originated forms of books for appointments, daily records and ledger use. These persons, for the sole purpose of profit, have done almost entirely the educational work for our profession along that line, and this is well so far as it goes.

This is a progressive age. The fourth quarter of the outgoing century is claimed to have been the greatest age of progress the world has ever known. That glory seems well given, for her strides in sciences, mechanics and the professions have been colossal; but at the same time there is perhaps no age and no nation whose life and activities are so inseparably connected with the commercial spirit of the times as this same age.

Commercialism rules the day. Professions, businesses, trades, progress in mechanics and to some extent in scientific discoveries, get their life-blood from the commercialism that infects the present age. It is a fact undeniable that we as a class are in our profession for the money we can get out of it. I have trained somewhat with professional brothers who claimed they were in the profession for the love of it and the love of humanity, but I am too well acquainted with human nature to let that virile arrow from their professional quiver pierce even the corneous layer of the epithelium of my being; and, if called upon to express myself in legal language, I would say they were guilty of false pretense, and in the vulgar language of the day it could be still more forcibly expressed.

I would say of myself, and I think it will apply to all to those self-spoken martyrs to the good of humanity, that I am always willing and over-glad to give my best services and the best of my knowledge and attainments, but I usually let my patients know that I live thereby and that the fee is a great consideration. Nor do I cast any odium on the members of the profession for their attitude, but to give one's services and charge a double or triple fee, then claim that the fee is no object but that the motive is a sacrifice for humanity, is the zenith of inconsistency and barefaced hypocrisy.

I agree with the generally established code of professional ethics only so far as it is practical and practiced, but to me the above practice, if not beyond the pale of our code of ethics, is certainly beyond our code of morals.

It is a fact universally admitted that a professional man is seldom a business man. This is evident in our own profession, for while we render invaluable services to mankind, as a rule we never get rich, and few get beyond a dependence on daily toil for their bread. The one seems to spoil him for the other, or else only those with no natural business talent enter the professions. The latter reason is somewhat strengthened by the statement of numerous people to us that they "would not be a dentist for anything."

This is sufficient reason for the study and discussion of the business part of our profession. It is the most practical part, for on it depends our success and the extent of our usefulness as dentists.

The first bookkeeping I ever did in my profession was by a system of figures and signs whereby I could tell the tooth I filled and the kind of filling, but not the location of the filling in the tooth. This, as my experience taught me, was unsatisfactory and unreliable. In my next system I used the old style pocket appointment book which was ruled for the hours for six days in the week. This book contained no place for remarks or addresses except in the back of the book, and contained no place whatever for a record of the work done. For this purpose I used a slate and made after each sitting a note of my work thereon, and at night would enter it in my ledger. The ledger I used was the Allport, the only one to my knowledge sold at that time. This gives diagrams which are very incomplete, as not all fillings can be located thereon. I was dissatisfied with that method, and, being unable to find any which approximated my ideal extant at that time, I resolved to embody my own ideas in a book for dental records and accounts. After three years and three different books, I have a form which simplifies the work more than any other with which I am acquainted.

Bookkeeping for a dentist should embrace all the records from that on the examination blank to the ledger account, the collection of the bill and the balancing of the account, and should not stop there, for the preservation of all those records is a matter of great importance.

No dentist's bookkeeping is complete unless his method makes a record of all the above, plain, simple, exact and reliable, and so they can be understood by his patients. To my knowledge this is done by very few dentists.

The systems that come nearest to it are Trigg's system of dental charts, Dart's system of books, and a method of my own devising. In the Trigg's system I find much to admire and praise, but its shortcomings seem to me to be in the fact that it has no way of preserving a record of appointments, it has no day book, and to review one day's work would require much labor, and it is expensive and in time cumbersome.

The Dart system, which has been widely advertised to the profession, has good points in the manner of keeping appointments and recording the work done. All work is recorded in connection with the appointment as made in the book, and the book is a complete appointment and day book. It also has a form of diagram in which very little improvement could be made. In this system a ledger is not exactly a necessity, but is certainly a great convenience.

Besides these the Allport ledger, which is liked for its simplicity, is yet in use, but to me its simplicity is its fault. Also the Warren dental ledger, whose diagrams are nearly faultless, is extensively used as a diagrammed ledger, but this must be preceded by one or two other books.

My own system, which contains advantages not found in any other, is complete in two books. My appointment, day book and cash book are combined in one. It is ruled for half-hour appointments and for recording the work done, the kind of work, the charge, amount paid and the expenses of the day, and each day's space contains diagrams on which a filling in any location may be plainly and definitely located. It is ruled for seven days in the week. When the book is open the whole week's space is before your eyes and also ample space for addresses, descriptions of unusual operations, remarks, etc. When you turn one leaf more you have diagrams for all of the following week before you. Turn one leaf back and the record of the previous week is before you. It is made in size for one year's records.

I have as yet no ledger made to correspond, but any diagrammed ledger or any ordinary double column business ledger can be used. My procedure is as follows: I use the ordinary examination blank, make my examinations with kinds of filling, price and date and file for reference until work is complete and bill paid. In my appointment and day book I make the appointment in the proper place. When the time comes, if the patient is there I do the work, and as soon as done I sit down and diagram the work, designate the kind, the charge and amount paid on the line with the appointment. If the patient does not come I mark it "ap. bro." for appointment broken; if from sickness I mark it so, and if previously changed I mark it so. I take the patient's address, and that, with any remarks, I record in its proper place without turning a leaf of the book. Every week or so I post my accounts in the ledger. At present I am using the ordinary double column business ledger. This is all that is necessary, for I have my diagrammed record in my day book, and my ledger date tells the exact page of the day book on which the work is recorded. My ledger is only for keeping totals and settling accounts. At the end of a year I have a complete year book which gives a complete record of my appointments, kept or broken, and why; a complete record of each day's work, charges, cash, outlay and addresses and remarks, all in their proper connection. In this book I have a record of all treatments and operations however slight, in their minutiæ.

The treatments are not all posted in the ledger, thus saving time and space. I have never seen another system so simple or complete in this particular.

So much for keeping accounts, but the end does not come until they are collected. I can only touch on this, or lack of time. We all know that a new man in the profession has the largest practice of any among professional deadheads. This is an experience we all pass through, but the experience is good schooling. For the past five years I have collected fully 98 percent of my earnings.

How did I do it? First, I quit working for strangers who came without reference or who could not satisfy me as to their responsibility. I usually ask a stranger before or during the first sitting who referred him to me. If they could not give me a satisfactory answer I would give them to understand I required cash.

I have had hanging for several years in front of my operating chair a sign, "Terms cash at each sitting unless otherwise previously arranged."

Does not this drive away patients? Perhaps so, but those who are reliable and of honest intent will take no offence at that, and they who do are welcome to go, and any brother dentist who is willing to take them and say nothing about pay is welcome to them.

I openly confess that I do not like to work for nothing, and I would rather lose one good patient with a dozen bad ones than to take the whole thirteen with the trouble, worry and loss.

This is another thing in which dentists are far behind other business men of the country. Hardly any business or community but whose members are commercially rated, and dealers can find beforehand the standing of any person. Such ought to be the case with the dentists of every town and city. They should have a protective association for the purpose of listing for their private use all persons known by any of them to deadhead their dental bills, this list to be revised and printed every six months and kept at hand for reference.

Information of this association should be printed in the town papers, and probably on billheads, showing its objects and its workings. The people would soon understand it, and the undesirable would not present themselves without the money to pay cash for their work. After giving this considerable thought for a few years, I am convinced it can be legally done, and would benefit not only the dentists but the community at large.

But time, or the lack thereof, forces me to close this paper by saying that the best way to collect accounts is to make no uncollectible accounts.

